# Safely Treating a Pulmonary Embolism in a Patient With Hereditary Hemorrhagic Telangiectasia: A Case Report

**DOI:** 10.1002/ccr3.72750

**Published:** 2026-05-27

**Authors:** Christina Carfagnini, Manasa Kandula

**Affiliations:** ^1^ Department of Internal Medicine University of Illinois Peoria Peoria Illinois USA

**Keywords:** anticoagulants, arteriovenous malformations, hereditary hemorrhagic, telangiectasia, venous thromboembolism

## Abstract

Hereditary Hemorrhagic Telangiectasia (HHT) is a rare autosomal dominant bleeding disorder. The incidence of venous thromboembolisms among HHT patients is significantly greater than the general population. However, providing therapeutic anticoagulation in patients with an increased propensity for bleeding creates a clinical dilemma. This is a case of a 58‐year‐old Caucasian female with a past medical history of HHT who was diagnosed with acute on chronic bilateral pulmonary embolisms. She was started on therapeutic anticoagulation after thoroughly considering the individual bleeding risks. She later developed upper gastrointestinal bleeding secondary to angioectasias in the stomach and duodenum that were treated with argon plasma coagulation. The patient completed a total of 6 months of therapeutic anticoagulation and another year of lower dose anticoagulation for secondary prevention. This case exemplifies that patients with HHT may be treated with anticoagulation after thorough risk benefit discussions and close monitoring for acute blood loss, as recommended by current guidelines. However, these guidelines are informed by low quality evidence and there is a lack of detailed data reported in current literature. Future studies directly comparing the safety of different anticoagulants among HHT patients are needed to inform clinical practice.

## Key Clinical Message

Hereditary hemorrhagic telangiectasia (HHT) patients may be treated with anticoagulation when indicated. Guidelines require thorough risk benefit discussions and close monitoring for acute blood loss. However, these guidelines are informed by low quality evidence and there is a lack of detailed data currently reported.

**Abbreviations**
APC: argon plasma coagulation
AVMs: arteriovenous malformations
DOACs: direct‐oral‐anticoagulants
ED: Emergency Department
EGD: esophagogastroduodenoscopy
GIB: gastrointestinal bleeding
HHT: hereditary hemorrhagic telangiectasia
LMWH: low molecular weight heparin
LUL: left upper lobe
PE: pulmonary embolism
pRBC: packed red blood cells (pRBC)
RLL: right lower lobe
VKA: vitamin K antagonists
VTE: venous thromboembolism

## 1 Introduction

Hereditary hemorrhagic telangiectasia (HHT) is a rare autosomal dominant bleeding disorder. HHT is diagnosed according to Curaçao's criteria. These criteria include recurrent epistaxis, mucocutaneous telangiectasias, visceral arteriovenous malformations (AVMs), and a first‐degree relative diagnosed with HHT [1, 2]. HHT is caused by mutations in *ENG* and *ACVRL1* genes that alter TGFβ signaling and regulate angiogenesis [1, 2]. HHT affects 1 in 5000 patients [1]. Clinicians need to be familiar with guidelines for managing co‐morbidities in this patient population, as it is not unlikely to come across HHT patients in their practice.

It is especially important to be familiar with guidelines for therapeutic anticoagulation in HHT patients. Despite having an increased risk of bleeding, HHT patients have no intrinsic protection against venous thromboembolism (VTE) because the underlying pathology is due to a vascular abnormality. The incidence of VTE among HHT patients was 138.3 per 100,000 person‐years, which was significantly higher than the general population [3]. The higher incidence of VTE has been attributed to low iron levels leading to increased transcription of factor VIII and von Willebrand Factor, resulting in shortened activated partial thromboplastin time [3]. In a study done on the HHT population, the degree of factor VIII elevation correlated with their thrombotic risk [4]. Treating VTE with therapeutic anticoagulation in a patient population with an increased propensity for bleeding creates a clinical dilemma that warrants individualized risk–benefit discussions.

## 2 Case History/Examination

A 58‐year‐old Caucasian female with a past medical history of HHT presented to the emergency department (ED) with sharp right‐sided flank pain. This patient was known to have all four Curaçao's criteria. Manifestations of HHT in this patient included a left upper lobe (LUL) pulmonary AVM that was embolized 2 years prior. In addition, multiple central nervous system AVMs were treated with gamma‐knife radiosurgery and residual AVMs excised via craniotomy 6 years ago. The patient was heterozygous for a pathological frameshift mutation in the ENG gene. Epistaxis and other HHT‐related symptoms were stable. The patient had a baseline hemoglobin of 10 g/dL and was not iron deficient on her most recent set of laboratory investigations 6 months prior.

**FIGURE 1:**
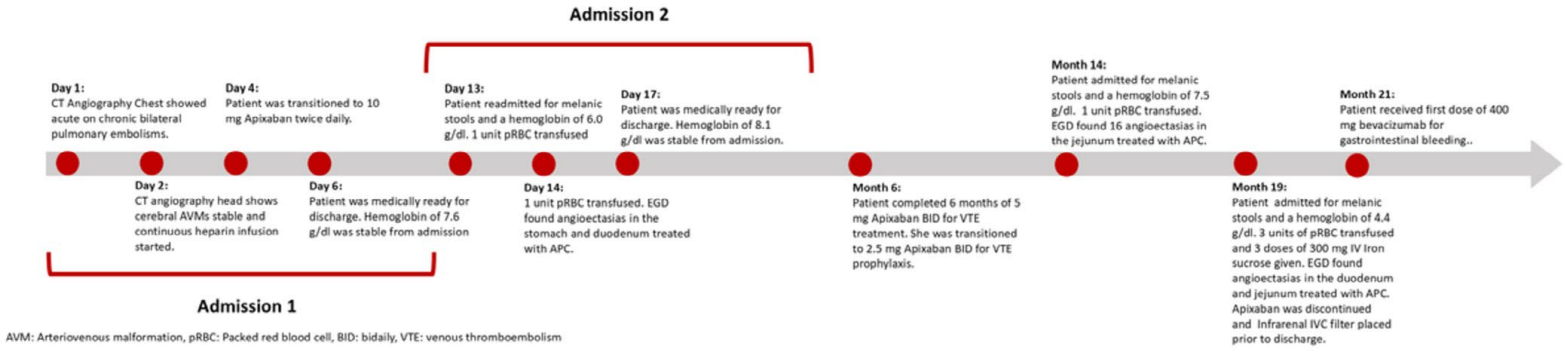
Timeline of 58‐year‐old patient with a past medical history of hereditary hemorrhagic telangiectasia admitted for a pulmonary embolism treated with therapeutic anticoagulation. Clinical course was complicated by a gastrointestinal bleed that was managed conservatively.

The patient was afebrile and hemodynamically stable upon admission, which is illustrated in Figure 1. Her saturation of peripheral oxygen was 90% with two liters of nasal cannula, although the patient did not require supplemental oxygen at baseline. Physical exam was otherwise unremarkable.

## 3 Differential Diagnosis, Investigations and Treatment

**FIGURE 2:**
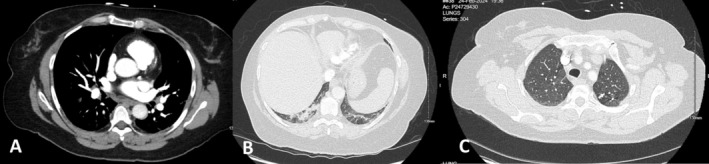
Computed Tomography Angiography visualizing bilateral pulmonary embolisms (A), right lower lobe pulmonary infarct (B), and a left upper lobe pulmonary arteriovenous malformations status post coiling (C).

A computed tomography (CT) abdomen‐pelvis done to evaluate the flank pain showed a pulmonary infarct in the right lower lobe (RLL) of the lung. A Well's score was zero but based on the pulmonary infarct finding and known increased incidence of VTE in patients with HHT, a dedicated CT angiography chest was done (Figure 2). This showed acute on chronic bilateral pulmonary embolisms (PEs) and a RLL pulmonary infarct. Appearance of the LUL pulmonary AVM was unchanged from prior imaging. The patient was diagnosed with low‐risk PE given serial troponins were less than 4 ng/L, pro‐ brain natriuretic peptide was 162 pg/mL and right heart strain was not seen on transthoracic echocardiogram or CT angiography. Of note, laboratory values are summarized in Table 1. This aligned with a low risk Class II Pulmonary Embolism Severity Index of 78 points. The patient was also found to have multiple left deep venous thrombi on duplex ultrasound. These VTEs were considered unprovoked given no history of recent surgery or prolonged travel, no tobacco or hormonal medication use, and all routine cancer screening was up to date. She also had no family history of clotting disorders. Hypercoagulable workup was unremarkable, including active partial thromboplastin time—lupus sensitive (32.4 s). Elevations in anticardiolipin IgM (23.6 U/mL) and β2 glycoprotein 1 (22.2 U/mL) were not sufficient to meet criteria for antiphospholipid syndrome.

**TABLE 1:** Summary of laboratory values.

	Laboratory result	Value
Baseline	Hemoglobin	10.0 g/dL
Admission 1	Troponin	> 4 ng/L
	Pro‐ brain natriuretic peptide	162 pg/mL
	pTT—Lupus sensitive	32.4 s
	Anticardiolipin IgM	23.6 U/mL
	β2 Glycoprotein 1	22.2 U/mL
Admission 2	Hemoglobin	6.0 g/dL
Month 14	Hemoglobin	7.5 g/dL
Months 19	Hemoglobin	4.4 g/dL

Abbreviations: Pro‐BNP, pro‐brain natriuretic peptide; pTT—Lupus sensitive, active partial thromboplastin time—lupus sensitive.

Anticoagulation was started after thoroughly considering the individual bleeding risks. Neurosurgery was consulted to assess the risk of intracranial bleeding given her history of cerebral AVMs. A CT angiography head with and without contrast showed changes associated with previously treated AVMs were stable from past imaging. After multidisciplinary evaluation and shared decision‐making with the patient, the risks and benefits of anticoagulation for acute venous thromboembolism were carefully reviewed. Discussion included the high mortality associated with untreated pulmonary embolisms and the risk of VTE progression with related morbidity, balanced against the increased bleeding risk in hereditary hemorrhagic telangiectasia. Her previously treated cerebral and pulmonary arteriovenous malformations were stable, and she had no history of previous gastrointestinal AVMs, major gastrointestinal or other life‐threatening bleeding. Given this clinical context, therapeutic anticoagulation with unfractionated heparin was initiated, as the anticipated thrombotic benefit was judged to outweigh the risk of hemorrhage. Vitamin K antagonists (VKA), low molecular weight heparin (LMWH), and direct‐oral‐anticoagulants (DOACs) were discussed with the patient given similar rates of bleeding in HHT patients [5, 6]. The hematology team transitioned the patient to 10 mg apixaban twice daily, as DOACs would not interact with her antiepileptic medications. DOACs were chosen over subcutaneous LMWH due to patient preference and convenience. She tolerated anticoagulation without epistaxis or gastrointestinal bleeding (GIB) for a total of 5 days prior to discharge, which included 2 days of DOACs. She was discharged home on therapeutic anticoagulation and supplemental oxygen.

## 4 Conclusion and Results

The patient presented to the ED 8 days later with black tarry stools. She was hemodynamically stable and with a hemoglobin of 6.0 g/dL that was notably lower than her baseline. Two units of packed red blood cells (pRBC) were transfused. Esophagogastroduodenoscopy (EGD) found angioectasias in the stomach and duodenum that were not seen on past EGD from 2020. Angioectasias were treated with argon plasma coagulation (APC). After cessation of bleeding and treatment of angioectasias with APC, apixaban was resumed at discharge. She also completed a left lower extremity venous duplex ultrasound and repeat anti‐phospholipid and lupus anticoagulant levels 3 months later, which were unremarkable.

The patient tolerated 6 months of therapeutic anticoagulation for treatment of an initial unprovoked VTE. Although current guidelines support extended anticoagulation beyond 6 months for unprovoked pulmonary embolism, her elevated bleeding risk related to vascular malformations was carefully considered. After completing 6 months of therapeutic anticoagulation, the regimen was de‐escalated to apixaban 2.5 mg twice daily for extended secondary prevention. However, she was hospitalized twice for melena at 14 and 19 months after her initial episode of PE. The degree of anemia in the latter admission was severe at 4.4 g/dL, requiring 3 units of pRBCs as well as three doses of 300 mg of intravenous iron sucrose. EGD at that time again revealed multiple angioectasias in the duodenum and jejunum that were treated with APC. Given the increasing frequency and severity of gastrointestinal bleeding, anticoagulation was discontinued, and an inferior vena cava filter was placed before discharge. After cessation of oral anticoagulation, bevacizumab therapy was initiated to reduce further HHT‐related gastrointestinal bleeding.

## 5 Discussion

Managing VTE in patients with HHT presents a significant challenge due to the paradox of high bleeding risk coexisting with the elevated thrombotic risk. This patient was managed according to the second international guidelines for the diagnosis and management of HHT. These guidelines recommend therapeutic anticoagulation when indicated while considering the individual's bleeding risks [1]. However, the quality of evidence for this recommendation is low. The two studies cited in these guidelines utilize self‐reported surveys that raise the potential for recall bias [7, 8]. A review investigating anticoagulation and antiplatelet therapy in HHT patients could not challenge or support this recommendation due to the lack of detailed data reported in the literature [2].

This case exemplifies that patients with HHT require a highly individualized and multidisciplinary approach and may be treated with anticoagulation after thorough risk benefit discussions and close monitoring for acute blood loss. Previous case reports of VTE in HHT patients are summarized in Table 2 [5, 6, 9]. This is the first case of an HHT patient with a VTE treated with DOACs. This patient experienced non‐fatal GIB with gastric and duodenal AVMs while on apixaban. These locations are the most common sites for gastrointestinal AVMs among HHT patients. Several studies found GIBs are the second most common adverse effect of anticoagulation among HHT patients, which is preceded by worsening recurrent epistaxis [2, 10]. This patient completed 6 months of therapeutic anticoagulation after pRBC transfusions and APC of AVMs. This aligns with prior case reports that HHT patients tolerated 6 months of therapeutic anticoagulation for initial unprovoked VTE when treating bleeding symptoms. Although AVMs were not visualized on the patient's prior EGD, it is important to consider that submucosal or microscopic AVMs may not be appreciated on intraluminal studies. The visualization and diagnosis of AVMs may also improve with new higher resolution endoscopy tools. It is important to monitor for GIBs in all HHT patients on therapeutic anticoagulation regardless of past EGD findings. The patient did not experience an intraparenchymal brain bleed while on therapeutic anticoagulation despite her extensive history of central nervous system AVMs. This observation aligns with current literature reporting major bleeding events are rare [8]. Although it should be noted that our patient's central nervous AVMs were adequately treated, risk was appropriately assessed before initiating anticoagulation. This may also be related to the use of apixaban instead of warfarin, as previous studies have shown decreased intracranial hemorrhage with DOACs as compared to warfarin in patients with non‐valvular atrial fibrillation [11].

**TABLE 2:** Summary of anticoagulation strategies used for venous thromboembolisms in hemorrhagic hereditary telangiectasia patients from previously published case reports.

Author, year	Age (Years)	Sex	VTE	Anticoagulant	Duration of AC (months)	Bleeding events	Outcome	Notes
Naeem 2025 [9]	84	F	PE	LMWH	10	Epistasis, GIB	Mortality	Family transitioned to comfort care as patient was unable to be resuscitated with blood products due to religious beliefs.
Serra 2015 [6]	74	F	PE, DVT	LMWH	6	Epistasis	AC discontinued	AC was discontinued upon completion of VTE treatment.
Finsterer 2015 [5]	64	NR	DVT	Phenprocoumon (VKA)	16	GIB	Mortality	Patient developed multiple thrombosis and bleeding events while on a VKA not approved by the FDA.

Abbreviations: AC, anticoagulation; F, female; FDA, food and drug administration; GIB, gastrointestinal bleed; LMWH, low molecular weight heparin; NR, not reported; VKA, vitamin K antagonist.

High‐quality studies comparing the safety of anticoagulant agents among HHT patients are needed to inform clinical guidelines. Current guidelines recommend therapeutic LMWH or VKA, due to readily available reversal agents and laboratory parameters to monitor therapeutic levels [1]. Such practice may subject patients to subcutaneous injections, laboratory monitoring and drug interactions without evidence supporting a clinical benefit, as summarized in Table 3. Studies investigating the safety of therapeutic anticoagulation among HHT patients do not compare different classes of anticoagulation [5, 7, 12]. Other studies found that the incidence of epistaxis, GI bleeding, and rate of anticoagulation discontinuation was not significantly different between VKA, heparin, and DOACs [6, 10]. Only one study reported patient‐centered outcomes, such as the need for blood transfusions, hospitalizations, and discontinuing anticoagulation [13]. Future studies directly comparing the tolerability of DOACs, VKA, and heparin among HHT patients are needed. Future studies could also investigate the ideal dose and duration of anticoagulation in this patient population.

**TABLE 3:** Benefits and limitations of anticoagulation strategies.

	Benefits	Limitations
Low molecular weight heparin	Easily available reversal agents Does not require drug monitoring.	No oral formulation available.
Vitamin K antagonists	Oral formulation Easily available reversal agents	Requires monitoring of INR Multiple drug–drug interactions
Direct oral anticoagulants	Oral formulation Does not require drug monitoring.	No easily available reversal agents

This is a rare case of an HHT patient receiving therapeutic anticoagulation for VTEs after considering the patient's individual bleeding risks, as recommended by current guidelines. The risk of bleeding from this patient's previously treated pulmonary and cerebral AVMs was considered lower than her risk of worsening VTE. She experienced upper GIB on apixaban, which exemplifies the need for close monitoring for acute blood loss irrespective of previous gastrointestinal AVM history. The GIB was treated symptomatically and the patient resumed anticoagulation for treatment of an initial unprovoked VTE. Multidisciplinary discussions and shared decision making are integral to the approach of this complex situation that is not infrequently encountered in this patient population. Future research investigating the safety of different anticoagulation agents, dosing and duration of anticoagulation is needed to inform clinical practice.

## Author Contributions

**Christina Carfagnini:** conceptualization, resources, writing – original draft, writing – review and editing. **Manasa Kandula:** writing – review and editing.

## Funding

The authors have nothing to report.

## Ethics Statement

The authors have nothing to report.

## Consent

The patient provided written informed consent for this publication. The patient received a signed copy of the consent form from the authors' institution.

## Conflicts of Interest

The authors declare no conflicts of interest.

## Data Availability

The authors have nothing to report.
